# Different Safety Pattern of an Inactivated SARS-CoV-2 Vaccine (CoronaVac^®^) According to Age Group in a Pediatric Population from 3 to 17 Years Old, in an Open-Label Study in Chile

**DOI:** 10.3390/vaccines11101526

**Published:** 2023-09-26

**Authors:** Nicole Le Corre, Katia Abarca, Patricio Astudillo, Marcela Potin, Sofía López, Macarena Goldsack, Vania Valenzuela, Andrea Schilling, Victoria Gaete, Lilian Rubio, Mario Calvo, Loreto Twele, Marcela González, Daniela Fuentes, Valentina Gutiérrez, Felipe Reyes, Lorena I. Tapia, Rodolfo Villena, Angello Retamal-Díaz, Antonio Cárdenas, Eduardo Alarcón-Bustamante, Xing Meng, Qianqian Xin, José V. González-Aramundiz, María Javiera Álvarez-Figueroa, Pablo A. González, Susan M. Bueno, Jorge A. Soto, on behalf of the PedCoronaVac03CL Study Group, Cecilia Perret, Alexis M. Kalergis

**Affiliations:** 1 Departamento de Enfermedades Infecciosas e Inmunología Pediátricas, División de Pediatría, Escuela de Medicina, Pontificia Universidad Católica de Chile, Santiago 8330077, Chile; mlec@uc.cl (N.L.C.); kabarca@uc.cl (K.A.);; 2 División de Pediatría, Escuela de Medicina, Pontificia Universidad Católica de Chile, Santiago 8330077, Chile; 3 Clínica San Carlos de Apoquindo, Red de Salud UC Christus, Santiago 7610437, Chile; 4 Departamento de Pediatría, División de Pediatría, Escuela de Medicina, Pontificia Universidad Católica de Chile, Santiago 8330077, Chile; 5 Centro Médico San Joaquín, Red de Salud UC Christus, Santiago 7820436, Chile; 6 Departamento de Medicina Familiar, Escuela de Medicina, Pontificia Universidad Católica de Chile, Santiago 8330077, Chile; 7 Facultad de Medicina, Clínica Alemana Universidad del Desarrollo, Santiago 7610658, Chile; 8 Departamento de Pediatría, Clínica Alemana de Santiago, Santiago 7650568, Chile; 9 Servicio de Neonatología, Hospital Luis Tisné, Santiago 7910000, Chile; 10 Instituto de Pediatría, Universidad Austral de Chile, Valdivia 5110566, Chile; 11 Hospital Puerto Montt, Puerto Montt 5507798, Chile; 12 Facultad de Medicina y Ciencia, Universidad San Sebastián, Puerto Montt 5501842, Chile; 13 Hospital Dr. Gustavo Fricke, Viña Del Mar 2340000, Chile; 14 Departamento de Pediatría, Universidad de Valparaíso, Valparaíso 2361845, Chile; 15 Unidad de Infectología Pediátrica, Servicio de Pediatría, Complejo Asistencial Dr. Sótero del Río, Santiago 8150215, Chile; 16 Departamento de Pediatría y Cirugía Infantil Norte, Hospital Roberto del Río, Facultad de Medicina, Universidad de Chile, Santiago 8380418, Chile; 17 Programa de Virología, Instituto de Ciencias Biomédicas, Facultad de Medicina, Universidad de Chile, Santiago 8380453, Chile; 18 Hospital Exequiel González Cortés, Facultad de Medicina, Departamento de Pediatría y Cirugía Infantil Campus Sur, Universidad de Chile, Santiago 8900085, Chile; 19 Departamento de Biotecnología, Facultad de Ciencias del Mar y de Recursos Biológicos, Universidad de Antofagasta, Antofagasta 1271155, Chile; 20 Hospital Clínico Universidad de Antofagasta, Universidad de Antofagasta, Antofagasta 1270001, Chile; 21 Millennium Institute on Immunology and Immunotherapy, Santiago 8331150, Chile; 22 Departamento de Ciencias Médicas, Facultad de Medicina y Odontología, Universidad de Antofagasta, Antofagasta 1271155, Chile; 23 Servicio de Pediatría, Hospital Regional de Antofagasta, Antofagasta 1240835, Chile; 24 Faculty of Mathematics, Pontificia Universidad Católica de Chile, Santiago 7820436, Chile; 25 Millennium Nucleus on Intergenerational Mobility: From Modelling to Policy (MOVI) [NCS2021072], Santiago 7820436, Chile; 26 Interdisciplinary Laboratory of Social Statistics, Facultad de Matemáticas, Pontificia Universidad Católica de Chile, Santiago 7820436, Chile; 27 Sinovac Biotech, Beijing 100085, China; 28 Departamento de Farmacia, Facultad de Química y de Farmacia, Pontificia Universidad Católica de Chile, Santiago 7820436, Chile; 29 Facultad de Ciencias Biológicas, Pontificia Universidad Católica de Chile, Santiago 8330077, Chile; 30 Departamento de Ciencias Biológicas, Facultad de Ciencias de la Vida, Universidad Andrés Bello, Santiago 8370251, Chile; 31 Departamento de Endocrinología, Facultad de Medicina, Escuela de Medicina, Pontificia Universidad Católica de Chile, Santiago 8330077, Chile

**Keywords:** CoronaVac^®^, SARS-CoV-2, COVID-19 vaccines, inactivated virus vaccine, safety pattern, children, adolescents

## Abstract

During the COVID-19 pandemic, the importance of vaccinating children against SARS-CoV-2 was rapidly established. This study describes the safety of CoronaVac^®^ in children and adolescents between 3- and 17-years-old in a multicenter study in Chile with two vaccine doses in a 4-week interval. For all participants, immediate adverse events (AEs), serious AEs (SAEs), and AEs of special interest (AESIs) were registered throughout the study. In the safety subgroup, AEs were recorded 28 days after each dose. COVID-19 surveillance was performed throughout the study. A total of 1139 individuals received the first and 1102 the second dose of CoronaVac^®^; 835 were in the safety subgroup. The first dose showed the highest number of AEs: up to 22.2% of participants reported any local and 17.1% systemic AE. AEs were more frequent in adolescents after the first dose, were transient, and mainly mild. Pain at the inoculation site was the most frequent AE for all ages. Fever was the most frequent systemic AE for 3–5 years old and headache in 6–17 years old. No SAEs or AESIs related to vaccination occurred. Most of the COVID-19 cases were mild and managed as outpatients. CoronaVac^®^ was safe and well tolerated in children and adolescents, with different safety patterns according to age.

## 1. Introduction

Since 2020, the COVID-19 pandemic has been responsible for millions of infected people and deaths worldwide [1]. By the end of 2020, more than 84 million people had confirmed COVID-19 and close to 2 million related deaths were reported to the World Health Organization (WHO) [2]. Older age, obesity, and immunocompromise were the main risk factors to developing a severe disease [3]. This led to the development of numerous therapeutic and preventive measures to mitigate its impact on morbidity, mortality, and economic repercussions [4]. Among these, vaccination became a critical tool for managing the pandemic. Since 2020, different platforms for SARS-CoV-2 vaccines have been proposed, such as mRNA, adeno-associated virus (AAV), and inactivated virus vaccines [5]. In adults, initial studies with these SARS-CoV-2 vaccines showed a 94%, 90%, and 84% efficacy to prevent symptomatic disease, respectively [6,7,8]. Moreover, they showed to be immunogenic and safe, with most adverse events (AEs) being mild and transient [9,10].

Vaccination was initially aimed at protecting the adult population; meanwhile, in children it was delayed considering their low incidence and low severity of COVID-19. However, over the course of the pandemic, complications of COVID-19, such as Multisystem Inflammatory Syndrome in Children (MIS-C), and severe illness in more susceptible children, became evident. This motivated the evaluation and subsequent use of the vaccine in this population [11,12]. Therefore, several clinical trials began to be carried out to evaluate the safety, immunogenicity, and efficacy of vaccines in the pediatric population [13]. mRNA, AAV, and inactivated SARS-CoV-2 vaccines showed a 91–100%, 98%, and 96% efficacy against COVID-19, respectively, in children and adolescents [14,15,16,17]. mRNA vaccines also showed a good safety profile with mild and transient AEs in children older than 5-years-old [14,16]. This data led to mRNA SARS-CoV-2 vaccines being approved by the U.S. Food and Drug Administration (FDA) in adolescents from 12 years and older and for emergency use in children from 6 months [18]. The European Medicines Agency (EMA) has authorized the use of mRNA vaccines in children older than 6 months [19].

The use of inactivated SARS-CoV-2 vaccines was interesting due to the vast knowledge of this platform regarding safety. Several studies with the inactivated SARS-CoV-2 vaccine CoronaVac^®^ (Sinovac Life Sciences, Beijing, China) in adults have reported a very good safety profile, local pain being the most frequent AE, mild systemic AEs, very low frequency of fever, less AEs in >60 years old adults and no SAEs related to the vaccine [20,21,22]. With this data, the WHO recommended the use of this vaccine in adults [23]. In Chile, given the safety profile and immunogenicity data, the Chilean Ministry of Health (MoH) authorized the use of Coronavac^®^ in adults in January 2021. Contrasting with this good safety profile, post-marketing surveillance described serious AEs such as thrombotic events and myocarditis associated with the AAV and mRNA vaccines, respectively [24,25].

To assess the safety and immunogenicity of the inactivated SARS-CoV-2 vaccine CoronaVac^®^ (Sinovac Life Sciences, Beijing, China) in children, a phase 1/2 clinical trial was performed with 72 and 480 children between 3 and 17 years old, respectively [15,26]. The vaccine was safe, with mild AEs; local pain was the most frequently reported AE compared to the placebo. Fever was the most common systemic AE. Interestingly, adolescents presented more AEs than younger children [15]. 

A multi-center international phase 3 clinical trial evaluating the efficacy, safety, and immunogenicity of CoronaVac^®^ in children and adolescents was developed. In Chile, because of the early approval of this vaccine for the pediatric population by the MoH, this study was rapidly adapted to an open-label study focused on reactogenicity, safety, and immunogenicity, whose preliminary results were published by Soto et al. [27]. Here, from this open-label study, we report the reactogenicity and safety of two doses of CoronaVac^®^ in 3 to 17 years old children in Chile analyzing the data from the total population enrolled and followed up for 6 months after the second dose. Even though a short period of the placebo-controlled study in children 3–5 yo was performed, we consider it important to report in this manuscript the safety results of the vaccine compared to the placebo, which are shown in the Supplementary Material section.

## 2. Materials and Methods

### 2.1. Study Design and Participants

As the approval for the emergency use of CoronaVac^®^ by the Chilean MoH for children older than 5 years old, this study had to be open-label for participants from 6 to 17 years old and placebo-controlled only for children from 3 to 5 years old. Due to the extension of the Chilean MoH approval for the emergency use of CoronaVac^®^ for children 3 to 5 years old, the study had to be rapidly adapted to an open label for all ages. The study was designed to evaluate the reactogenicity, safety, and immunogenicity of two doses of CoronaVac^®^ in children and adolescents for six months after the second dose. In this report, we communicate the safety data, while the immunogenicity results are published elsewhere [27]. Participants were recruited from September 2021 to January 2022 in seven centers in Santiago and four in other cities: Viña del Mar (Center), Antofagasta (North), Valdivia, and Puerto Montt (South). The study was approved by the sponsoring institution Ethical Committee (ID 210616012), by the Ethical Committee of the other sites, and by the Public Health Institute of Chile (ISP Chile, number No. 20674/21), registered in Clinical Trials (NCT04992260), and conducted according to the current Tripartite Guidelines for Good Clinical Practices, Declaration of Helsinki, and local regulations [28].

**Figure 1:**
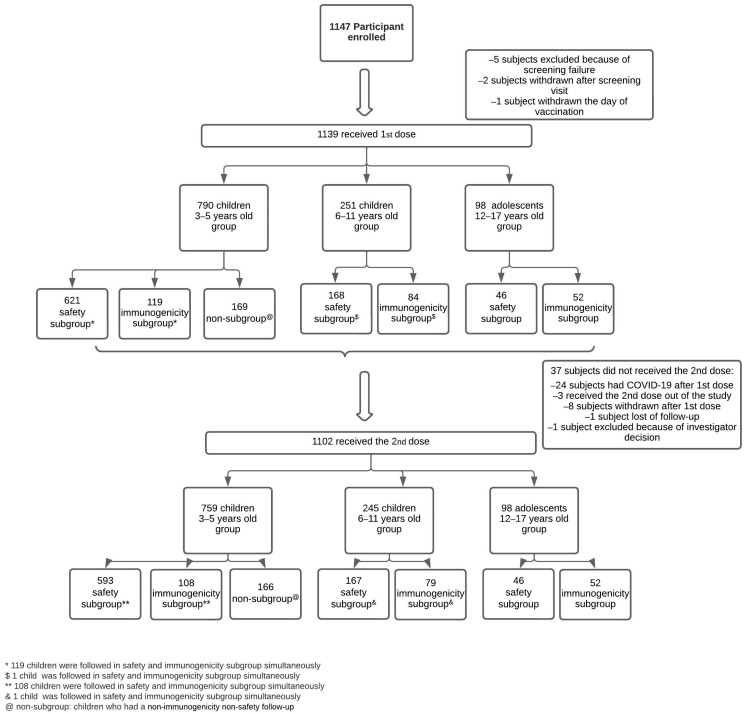
Study design flowchart: screening, randomization, and follow-up.

Written informed consent was signed by caregivers, and children older than 6 years old gave their assent before enrollment. After the inclusion criteria were met (Supplemental Material), participants were distributed into three subgroups according to the parental decision: Immunogenicity, to evaluate humoral and cellular responses at enrollment and at 1 and 6 months after the second dose. Safety, to follow up AEs in diary cards for 28 days after each dose. Non-immunogenicity–non-safety, to follow-up serious AEs (SAEs) and AEs of special interest (AESI) during all of the study, which were also followed up in the whole cohort (Figure 1). No other concomitant vaccines were allowed up to 7 days for inactivated and 14 days for attenuated organism-based vaccines, before and after the study vaccine. As it was mentioned before, children 3–5 years old were initially randomized into a double-blinded controlled placebo cohort. However, they were unblinded two months after the beginning of the study, and the participants were invited to continue in the open-label study. Those volunteers who received the placebo and decided to continue in the study were vaccinated with CoronaVac^®^ (Figure S1). After the unblinding, all new recruited children of 3–5 years old received the vaccine.

### 2.2. Procedures

Two doses of CoronaVac^®^, consisting of 3 µg of inactivated SARS-CoV-2 (600 SU/0.5 mL), in a 4-week interval, were administered to all participants divided into three age groups: 3–5, 6–11, and 12–17 years old [15]. The placebo, briefly used at the beginning of the study, consisted of 0.5 mL of aluminum hydroxide (Sinovac Life Sciences, Beijing, China).

Demographic information, comorbidities, concomitant medications, and nutritional status were obtained at enrolment and registered in an electronic case report form. A SARS-CoV-2 rapid antigen test from a nasopharyngeal swab and a SARS-CoV-2 IgM/IgG antibody rapid test (Cellex®, Morrisville, North Carolina, USA) were performed on all participants before vaccination. A urine pregnancy test was performed on all post-menarche or ≥13 years old girls. All participants were observed 30 min after each dose to evaluate possible immediate AEs. After vaccination, participants in the safety subgroup and their caregivers received a diary card and were instructed to register daily any local and systemic solicited AEs for 7 days after each dose and any other AEs (unsolicited) and concomitant medications until 28 days after the second dose. These cards were checked by investigators on day 7 and collected onsite on day 28. SAEs, AESI (described in the Supplementary Material), and relevant medications (immunosuppressed drugs, transfusions, and other vaccines) were collected from all participants for six months after the second dose by regular telephone calls. The severity of solicited and unsolicited AEs was graded using a scale of 1 to 4 and 1 to 5, respectively, based on the guidelines for the grading scale of AEs in vaccine clinical trials, 2019, of the National Medical Products Administration, China [29]. The causal relationship between AEs and vaccination was determined according to the classification adapted from the “Uppsala Monitoring Center” of the WHO [30].

During the brief period of the placebo-controlled study, those children had the same safety follow-up as those who received open-label CoronaVac^®^.

COVID-19 cases were monitored throughout the study via phone calls. If participants met the criteria for suspected COVID-19 cases (Supplementary Material), a respiratory sample was collected (nasopharyngeal swab or saliva) for SARS-CoV-2 RT-PCR. All confirmed symptomatic COVID-19 cases were followed up until recovery.

### 2.3. Outcomes

The primary safety outcomes were the occurrence of solicited and unsolicited AEs from the day of the first vaccination to 28 days after the second dose of the vaccine in the safety subgroup, the occurrence of immediate AEs post-vaccination, SAEs, and AESI in all vaccinated children throughout the study.

The secondary outcome was to describe the breakthrough COVID-19 cases, the occurrence of vaccine-enhanced disease (VED), defined as a severe disease or MIS-C cases, and the occurrence of hospitalization as a surrogate indicator of vaccine protection.

### 2.4. Statistical Analyses

Categorical variables were expressed as count and percentage while numerical variables as mean and standard deviation (SD) or median and range. For the comparison of averages, the *t*-test for independent samples was used. Categorical variables were evaluated using chi-square or Fisher exact test; the significance level was ≤0.05. The proportion of participants free of COVID-19 over time was estimated using Kaplan–Meier analysis, and age group curve differences were assessed using the log-rank test. All statistical analyses were performed using R version 4.2.0.

## 3. Results

### 3.1. Study Population

A total of 1139 children and adolescents between 3 and 17 years old were enrolled and received the first dose of CoronaVac^®^ and 1102 received the second one. Distribution by age and study subgroup is described in Figure 1. Information about the distribution of children 3–5 years old who were initially recruited in the vaccine/placebo study is available in Figure S1. Clinical and demographic baseline characteristics of vaccinated children are shown in Table 1. The mean follow-up time was 94.94 days after the first dose (range: 0–294 days) and 66.87 days after the second (range: 0–266 days). After the second dose, 1064 (97%) participants completed at least the 28-day follow-up. At enrollment, 36 subjects had previous COVID-19 diagnosed by detecting IgG/IgM (1 subject by IgM and 35 by IgG) and 4 had an asymptomatic infection documented by a rapid antigen test (Table 1).

### 3.2. Safety

Local immediate AEs were infrequent after vaccination. Out of all subjects, 6.5% presented at least one AE after the first dose and 3.9% after the second dose. Pain at the inoculation site was the most frequent local immediate AE reported in 3.6% of all subjects. AEs were observed in less than 2% (Table S1). Regarding immediate systemic AEs, all were reported at a very low frequency, less than 1% after the first and second doses, except headache in adolescents, occurring in 3% after the first dose (Table S2) and 2.5% after the second dose. Interestingly, in adolescents, immediate pain at the inoculation site increased from 1% to 10.2% after the second dose. 

**Figure 2:**
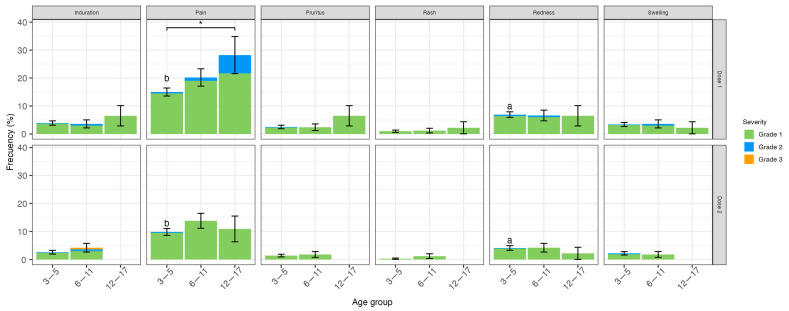
Local solicited adverse events after CoronaVac^®^ dose 1 and 2. Upper panel shows local AE frequency after dose 1 and lower panel after dose 2 for each age group (immediate local AEs are not included). Frequencies are expressed as a percentage; whiskers represent standard deviation. Colors represent grades of severity (green: mild, blue: moderate, orange: severe). * *p* ≤ 0.05. a and b: *p* ≤ 0.05 and *p* ≤ 0.01, respectively, comparison between doses.

**Figure 3:**
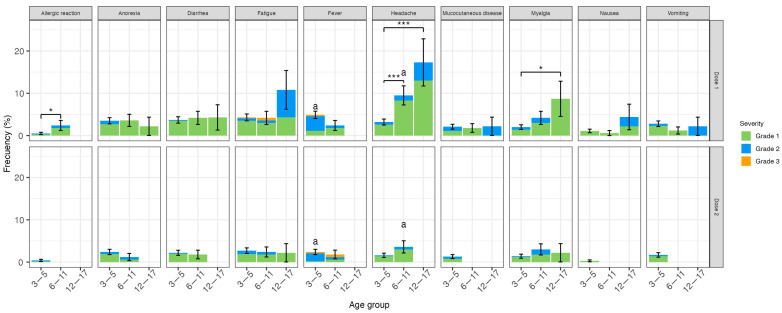
Systemic solicited adverse events after CoronaVac^®^ dose 1 and 2. Upper panel shows systemic AE frequency after dose 1 and lower panel after dose 2 for each age group (immediate systemic AEs are not included). Frequencies are expressed as a percentage; whiskers represent standard deviation. Colors represent grades of severity (green: mild, blue: moderate, orange: severe). * *p* ≤ 0.05, *** *p* ≤ 0.001; a: *p* ≤ 0.05 for comparison between doses.

In the safety subgroup, 835 children were followed up with diary cards after the first and 805 after the second doses (Figure 1). Figure 2 and Figure 3 show the local and systemic non-immediate solicited AEs, respectively. At least one local AE was reported in 22.2% of the participants after the first and 13.3% after the second dose. Pain at the inoculation site was the most frequent after both doses (between 15% and 28.2% after the first dose and between 9.8% and 13.8% after the second dose according to age group), having a significantly higher frequency in 12–17 years old than 3–5 years old (*p* = 0.03) after the first dose. The frequency of this AE diminished after the second dose, particularly in 3–5 years old (15% to 9.8%, *p* = 0.008). The incidence of redness also diminished significantly after the second compared to the first dose in 3–5 years old (6.9% to 4.1%, *p* = 0.04). All other local non-immediate AEs were reported in less than 7% of all vaccinated children and adolescents. Regarding systemic non-immediate AEs, 17.1% of subjects presented at least one AE after the first dose and 8.7% after the second dose. Headache was the most frequent after the first dose occurring in 5.3% of the subjects and being significantly higher in adolescents and 6–11 years old compared to 3–5 years old (*p* < 0.001 and *p* = 0.001, respectively, Figure 3). After the second dose, the headache was observed less frequently and, interestingly, was not seen in adolescents. Indeed, this reduction was significant for the 6–11 years old (9.5% to 3.6%, *p* = 0.05). Myalgia occurred more often in adolescents compared to 3–5 years old (*p* = 0.02) and allergic reactions in 6–11 years old compared to 3–5 years old (*p* = 0.04) after the first dose. Among the 9 children who had an allergic reaction after vaccination, 6 of them were classified as probable, 2 as possibly related, and 1 as non-related to the vaccination; all were mild to moderate in severity (Table S3). Fever was not reported among adolescents, but it was the most frequent systemic AE among 3–5 years old (4.9%) after the first dose but had a significant reduction after the second dose (2.4%), *p* = 0.02. All other systemic AEs were observed in less than 5% of all age groups, except fatigue which was the second AE more frequent in adolescents (10.8%) after the first dose. The severity of local and systemic AEs was Grade 1 (94.6% and 71.9%), Grade 2 (5.2% and 26%), and Grade 3 (0.2% and 2.1%), respectively. All AEs were transient. Local pain and myalgia had a median duration of 1 day (range <1–7 and <1–6 days, respectively), and fever, headache, and fatigue had a median of less than 1 day (range < 1–3, <1–8, and <1–5 days, respectively).

Regarding baseline SARS-CoV-2 infection, no statistical differences were observed in terms of the number of immediate AEs among the 40 positive subjects. In addition, 26 subjects belonging to the safety group had a previous history of COVID-19, and among them, no differences were observed concerning non-immediate solicited local and systemic AEs.

During the short period of the placebo-controlled trial, we were able to compare solicited AEs between CoronaVac^®^ and the placebo in 3–5 years old children. The frequency of local pain was significantly higher with CoronaVac^®^ compared to the placebo after the first dose (20.4% versus 11.4%, *p* = 0.03). No statistical differences were observed comparing other local or systemic AEs between both groups (Tables S4 and S5).

After the first dose, 483 unsolicited AEs were reported in 267 subjects (31.9%) and 869 in 342 (42.5%) subjects after dose 2. These events are described in Table S6. None of the participants discontinued the study due to an AE.

During this study period, 8 children reported SAEs described in Table S7. All children were hospitalized but all recovered completely. None of the SAEs were considered related to the vaccine. No AESI or VED were reported during follow-up. No use of relevant medications (immunosuppressed drugs and transfusions) was reported. However, 739 participants received other vaccines during the follow-up described in Table S8.

### 3.3. Breakthrough COVID-19 Cases

**Figure 4:**
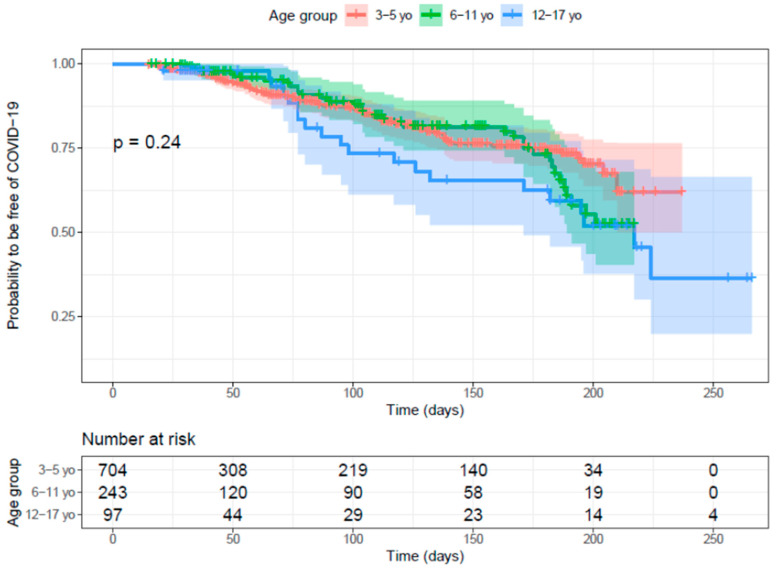
Time of SARS-CoV-2 infection after two doses of CoronaVac^®^.

During the follow-up period, 168 confirmed COVID-19 cases were detected. Among them, 135 occurred at least 14 days after the second dose. The interval of time between the vaccination and breakthrough cases is represented in Figure 4. No statistical differences were observed regarding sex, history of COVID-19, allergic rhinitis, or asthma between children with and without COVID-19 during the study. However, participants with confirmed COVID-19 were significantly older than those non-confirmed (7.1- [standard deviation (SD) = 3.90] and 6.2 [SD = 2.99]-years-old, respectively, *p* = 0.009). All COVID-19 cases were outpatients, except for one child with COVID-19 who was hospitalized due to a bacterial superinfection for three days requiring mild oxygen support. No cases of severe disease or MIS-C were observed.

The proportion of participants free of COVID-19 over time was assessed using Kaplan –Meier analysis, including 92 COVID-19 cases, and negative subjects with and without complete follow-up. Participants of 12–17 years old are in blue, green 6–11 years old, and red 3–5 years old; the color shade represents 95% CI. The table represents the number of participants at risk of COVID-19 over time. The placebo group was not included because of an early emergency use approval of vaccination in this age group by the MoH.

## 4. Discussion

Within the global efforts to provide SARS-CoV-2 vaccines to control the pandemic, we evaluated the safety and reactogenicity of an inactivated vaccine, CoronaVac^®^, in an immunocompetent pediatric population. Two doses of this vaccine showed an excellent safety profile, with local pain being the most frequent AE. Interestingly, different AE patterns were observed according to age group. However, most of the AEs were mild and all transient, lasting less than 48 h. During the follow-up, no SAEs or AESIs related to the vaccine were reported in this pediatric population.

The frequency of AEs after two doses of CoronaVac^®^ was consistent with the results of the previous phase 1 and 2 clinical trials, where AEs were present in less than 30% of children and where the pain in the injection site was the most frequently observed [15]. Recently, a large open-label study recruiting 31,041 children and adolescents conducted in China reported only 1.8% of AEs in children and adolescents [31]. This lower incidence of AEs may be explained by a different methodology used for reporting AEs: reporting was performed using a phone application or phone call, compared to our study, where a robust follow-up was carried out with self-filling cards and strict active surveillance. Although pain at the injection site was the most frequent AE in both studies, the results reported herein provide more details regarding systemic AEs, where different symptoms are observed depending on the age group evaluated. Interestingly, we observed fever more frequently in the youngest group and headache in the older group. Compared to the study carried out in adults, a similar frequency of local AEs was observed (26.6%), with local pain being the most frequent [20]. Regarding systemic AEs, as in the group of adolescents, headache was the most reported (26%), and fever was rarely reported, particularly in those over 60 years of age. Thus, adolescents show the same pattern of AEs as adults with an inactivated virus vaccine, while younger children display a different behavior, with fever and fatigue being particularly important in this age group. Regarding SAEs, no events related to vaccination were observed [15]. The present study confirmed these safety findings, with no vaccine-related SAEs after a mean follow-up time of 4 months in more than a thousand children.

Compared with other SARS-CoV-2 vaccine platforms, this study showed that CoronaVac^®^ has a notoriously lower frequency of local and systemic AEs, as recently shown in a meta-analysis [32]. A placebo-controlled blinded study showed that two doses of mRNA vaccine in adolescents were safe, but with 86% of local pain, 66% of fatigue, and 65% of headache [14]. The same was observed in children 5–11 years old with two doses of mRNA vaccine, showing a high frequency of local pain, reaching 74% of participants [16]. Among adolescents, 39% presented fatigue and 28% headache, more frequently after the second dose [16]. In our study, less than a quarter of CoronaVac^®^ participants reported any local AEs, with pain being the most frequent in 28% of the adolescents; and less than a fifth reported any systemic AEs, with headache being the most frequent in 18% of adolescents, after the first dose. Fever was observed in 40% of 6–17 years old children in an AAV vaccine trial [17], and in 24% of 6–11 years old children in a mRNA trial [33], contrasting with less than 3% in 6–11 years old and no cases in adolescents in our study. A particular AE pattern according to age was also described in an Australian post-marketing surveillance study, where a lower incidence of fever was observed in children receiving an mRNA vaccine, but with a higher frequency in adolescents as compared to children 5–11 years old (17% and 4.9% after the second dose, respectively) [34]. Interestingly, fever is nearly absent in adolescents in our study showing an important difference with vaccines from other platforms.

There is still scarce information about the safety and reactogenicity of SARS-CoV-2 vaccines in children up to 5 years old. In a recently published mRNA trial in this age group, local pain presented in 73%, fatigue in 48%, and fever in 16%, numbers that are much higher compared to CoronaVac^®^ in our study (up to 15% for local pain, and less than 5% for these systemic AEs) [35]. Regarding severity, all local AEs in this age group were mild to moderate in our study, while in the mRNA trial, a few cases of grade 3 were reported; in both studies, some cases of fever and fatigue reached grade 3, more frequently after the second dose in mRNA vaccines, in contrast to CoronaVac^®^ [35].

During the 6 months of follow-up, no SAEs or AESI such as myocarditis MIS-C related to the vaccine were reported in our study. The same was observed in two mRNA vaccine trials, evaluating 1500 children of 5–11 years old and in 3000 in 6–11 years old [16,33]. However, a post-authorization safety report and a recent meta-analysis of mRNA vaccines in children of 5–11 years old showed few cases of myocarditis after vaccination, estimating an incidence of 1.8–2.2 per 106 doses [36,37]. These findings contrast with the myocarditis incidence observed in adolescents, which reach 48 and 45.7 cases per 106 doses in two reports, from Israel and the USA, respectively [36,38]. Up to September 2022, no myocarditis cases post-CoronaVac^®^ vaccination in children less than 18 years old have been reported in the Chilean post-vaccine AE surveillance system [39]. However, as this event is rare, continuous surveillance Is needed.

Compared with the placebo, subjects receiving CoronaVac^®^ reported local pain more often; the same has been observed in mRNA clinical trials [35]. We observed no difference in systemic AE between vaccine and placebo recipients; in contrast, fever and fatigue were higher in mRNA vaccinated children 3 to 5 years old [35]. These observations would suggest that these mild AEs are reactions induced by the vaccine antigen, inactive SARS-CoV-2 virus, rather than by the adjuvant, aluminum hydroxide, present in both the vaccine and placebo.

The number of confirmed COVID-19 cases observed in this study is consistent with the large Omicron outbreak in Chile occurring during the follow-up of the participants [40]. Although there was a small number of infected children, no MIS-C or suspected VED cases occurred. Indeed, the incidence of MIS-C in Chile progressively decreased during the pandemic, coinciding with immunization coverage of children and adolescents in the country [41]. This same observation was highlighted in a multicentric study where vaccination was proposed as a tool for preventing MIS-C new cases [42]. Chilean studies evaluating CoronaVac^®^ effectiveness during this outbreak showed 75% of protection for COVID-19 and 94% for pediatric intensive care unit admission for children 6–16 years old and 38% and 69%, respectively, for children 3–5 years old [43,44]. This protection against severe COVID-19 cases is consistent with the humoral response induced by CoronaVac^®^ in this age group recently described by Soto et al. [27]. Although the antibody neutralizing capacity seems to decrease with the Omicron variant, the specific CD4 + T cell response was maintained [27]. Comparing the incidence of systemic symptoms in children with COVID-19 and CoronaVac^®^-related AEs is another way to weigh the risk/benefit of vaccinating children. A systematic review showed that COVID-19 symptoms are present in 85 to 60% of children and adolescents, mainly hospitalized, and fever is observed between 46 and 64% of cases [45]. This figures contrast with the low incidence of systemic AEs induced by CoronaVac^®^ in children, in favor of vaccination.

## 5. Conclusions

CoronaVac^®^ was shown to be safe in this pediatric population, with mainly mild and transient AEs and no SAEs or AESI related to the vaccine.

The frequency of the different solicited AEs depends on the age group. Pain is the main local AE in all age groups, but higher in adolescents. The main systemic AE is fever in young children, while headache is the main one above 5-years-old. AEs tended to be less frequent after the second dose. Notably, the frequency of AEs is lower than that observed in other platforms.

This safety profile, in addition to good effectiveness against severe disease, gives the confidence to continue developing this vaccine platform, particularly including new SARS-CoV-2 variants.

## Figures and Tables

**Table 1:** Clinical and demographic characteristics of 1139 vaccinated children and adolescents.

	3–5 Years Old Group *n* = 790	6–11 Years Old Group *n* = 251	12–17 Years Old Group *n* = 98
**Age years, mean** (**SD**)	4.5 (0.8)	8.7 (1.8)	13.6 (1.5)
**Gender female, *n*** (**%**)	392 (49.6)	117(46.6)	43 (43.9)
**Ethnicity**			
**Hispanic, *n***	713	233	94
**Chilean native, *n***	17	11	3
**White, *n***	22	4	0
**Others, *n***	38	3	1
**With co-morbidities**			
**Allergic rhinitis, *n***	150	31	8
**Asthma, *n***	51	8	5
**Atopic dermatitis, *n***	46	6	1
**Obesity, *n***	3	6	5
**ENT ^a^, *n***	5	2	0
**Mental health ^b^, *n***	15	11	3
**Drugs allergy, *n***	5	1	1
**Other allergies, *n***	18	1	4
**Metabolic–endocrine** **disorders, *n***	8	7	5
**Chromosomopathies ^c^, *n***	4	1	0
**Others, *n***	77	21	5
**Baseline SARS-CoV-2 positive status, *n*** (**%**) **^d^**	22 (2.8)	15 (6)	3 (3)

^a^ Ear, nose and throat; ^b^ anxiety disorders, ADHD, ASD, depression, feeding disorders; ^c^ Down Syndrome and Turner Syndrome; ^d^ Baseline SARS-CoV-2 positive status was defined as a positive rapid antigen test or a positive antibody test (IgM or IgG) at enrollment.

## Data Availability

The data are not publicly available due to ethical restrictions as all participants are deidentified.

## Supplementary Materials

The following supporting information can be downloaded at: https://www.mdpi.com/article/10.3390/vaccines11101526/s1: Figure S1: Flowchart vaccine/placebo study for 3- to 5-year-old children; Table S1: Immediate local adverse events after CoronaVac^®^ dose 1 and 2; Table S2: Immediate systemic adverse events after CoronaVac^®^ dose 1 and 2; Table S3: Description of solicited allergic reactions following vaccination; Table S4: Non-immediate solicited local adverse events in children 3–5-years-old receiving CoronaVac^®^ or placebo; Table S5: Non-immediate solicited systemic adverse events in children 3–5-years-old receiving CoronaVac^®^ or placebo; Table S6: Unsolicited adverse events reported during the study after CoronaVac^®^ dose 1 and 2; Table S7: Severe adverse events reported during the study after CoronaVac^®^ dose 1 and 2; Table S8: Other vaccine received during the study; Supplementary Methods: Inclusion and Exclusion Criteria, Adverse Events of Special Interest (AESI), COVID-19 case definition, Members of the PedCoronaVac03CL Study Group.
