# Supplementary material for: Different Safety Pattern of an Inactivated SARS-CoV-2 Vaccine (CoronaVac®) According to Age Group in a Pediatric Population from 3 to 17 Years Old, in an Open-Label Study in Chile
Source: Vaccines (Basel). 2023 Sep 26;11(10):1526. doi: 10.3390/vaccines11101526 (PMC10611329; doi:10.3390/vaccines11101526)
Supplement: Supplementary file 1 [file vaccines-11-01526-s001.zip › vaccines-2465282-Supplementary Material.pdf]

SUPPLEMENTARY MATERIAL:

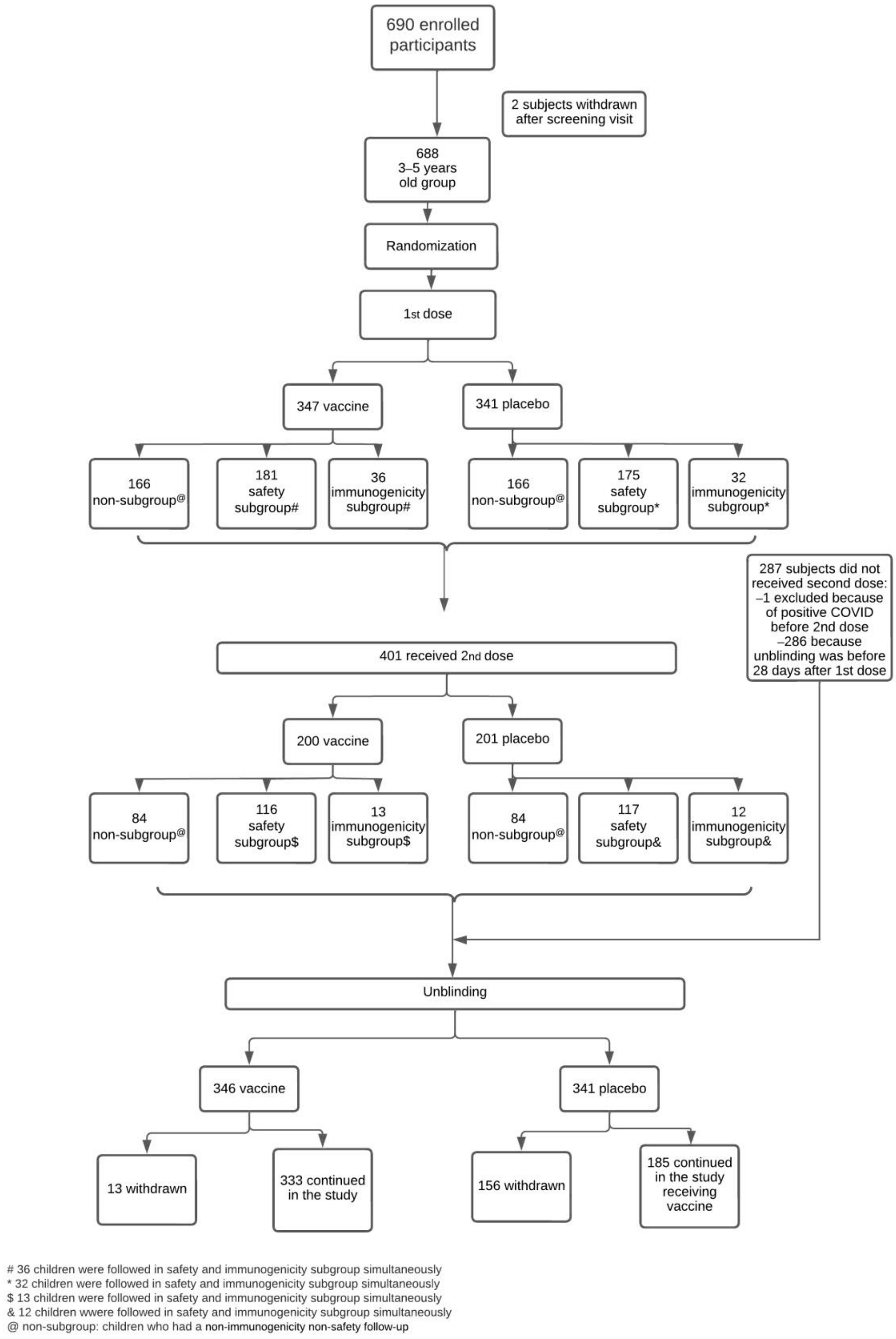

Figure S1: flowchart vaccine/placebo study for 3 to 5 years old children

Table S1: Immediate local adverse events after CoronaVac® dose 1and 2

Age (years)	Dose 1				Dose 2			
	G1	G2	G3	prop	G1	G2	G3	prop
**Pain**								
3-5	32	1	0	33/790	10	0	0	10/759
6-11	7	0	0	7/251	7	0	0	7/245
12-17	1	0	0	1/98	10	0	0	10/98
**Redness**								
3-5	18	1	0	19/790	13	0	0	13/759
6-11	2	0	0	2/251	1	0	0	1/245
12-17	0	0	0	0	0	0	0	0
**Induration**								
3-5	4	0	0	4/790	0	0	0	0
6-11	2	0	0	2/251	0	0	0	0
12-17	1	0	0	1/98	0	0	0	0
**Pruritus**								
3-5	1	0	0	1/790	1	0	0	1/759
6-11	3	0	0	3/251	0	0	0	0
12-17	0	0	0	0	1	0	0	1/98
**Swelling**								
3-5	0	0	0	0	0	0	0	0
6-11	0	0	0	0	0	0	0	0
12-17	1	0	0	1/98	0	0	0	0

G1, G2, G3: grade of severity 1 (mild), 2 (moderate), 3 (severe) respectively
Prop: proportion

Table S2: Immediate systemic adverse events after CoronaVac® dose 1and 2

Age (years)	Dose 1 G1	Dose 1 G2	Dose 1 G3	Dose 1 prop	Dose 2 G1	Dose 2 G2	Dose 2 G3	Dose 2 prop
**Fatigue**								
3-5	4	0	0	4/790	1	0	0	1/759
6-11	1	0	0	1/251	0	0	0	0
12-17	0	0	0	0	0	0	0	0
**Headache**								
3-5	1	2	0	3/790	0	0	0	0
6-11	3	0	0	3/251	1	0	0	1/245
12-17	3	0	0	3/98	1	0	0	1/98
**Myalgia**								
3-5	0	0	0	0	0	0	0	0
6-11	1	0	0	1/251	0	0	0	0
12-17	0	0	0	0	1	0	0	1/98
**Nausea**								
3-5	0	0	0	0	0	0	0	0
6-11	1	0	0	1/251	0	0	0	0
12-17	0	0	0	0	0	0	0	0
**Mucocutaneous Disease**								
3-5	1	0	0	1/790	0	0	0	0
6-11	1	0	0	1/251	0	0	0	0
12-17	0	0	0	0	0	0	0	0
**Vomiting**								
3-5	0	0	0	0	1	0	0	1/761
6-11	0	0	0	0	0	0	0	0
12-17	0	0	0	0	0	0	0	0

G1, G2, G3: grade of severity 1 (mild), 2 (moderate), 3 (severe) respectively
Prop: proportion

Table S3. Description of solicited allergic reactions following vaccination.

	**Age group**	**Allergic reaction**	**Timing**	**Severity**	**Classification by investigator**
Subject 1	6-11	Rash in her/his back	Three days after the 1st dose and last two days	Grade 1	Not related
Subject 2	6-11	This subject presented rash in face and arms	Three days after 1st dose, lasting one day without any medication	Grade 1	Probable
Subject 3	6-11	The subject has antecedents of atopy and has already facial rash before vaccination but an exacerbation of this rash in her/his face, trunk and arms occurred after vaccination	Three days after the 1st dose. The duration is unknown	Grade 2	Probable
Subject 4	6-11	Acute allergic reaction	Same day after the 1st dose. Lasting 1 day	Grade 1	Probable
Subject 5	3-5	Allergic reaction in the face, without dyspnea	One day after the 1st dose. Lasting 2 days	Grade 1	Probable
Subject 6	3-5	Generalized rash and urticarial plaques without angioedema, required treatment with antiallergics	Two days after the 2nd dose. Lasting 1 day	Grade 2	Possibly
Subject 7	3-5	Pruritus	Four days after the 2nd dose. Lasting 3 days	Grade 1	Probable
Subject 8	3-5	Pruritus in left arm	Seven days after the 1st dose. Lasting 1 day	Grade 1	Probable
Subject 9	3-5	Arms and legs redness	Five days after the 1st dose. Lasting 6 days	Grade 2	Possibly

Grade 1= mild
Grade 2= moderate

Table S4: Non-immediate solicited local adverse events in children 3-5 years old receiving CoronaVac® or placebo

Adverse event	CoronaVac® Dose 1 n=181		Placebo Dose 1 n=175		
	**n**	**prop**	**n**	**prop**	**p**
Pain	37	20.4	20	11.4	0.03
Redness	15	8.29	7	4	NS
Induration	10	5.52	5	2.86	NS
Swelling	9	4.97	3	1.71	NS
Pruritus	3	1.66	7	4	NS
Rash	0	0	1	0.57	NS
	**Dose 2 n=116**		**Dose 2 n=117**		
	**n**	**prop**	**n**	**prop**	**p**
Pain	12	10.3	9	7.69	NS
Redness	6	5.17	6	5.13	NS
Induration	4	3.45	0	0	NS
Swelling	4	3.45	0	0	NS
Pruritus	1	0.86	0	0	NS
Rash	0	0	1	0.85	NS

Prop: proportion; NS: no significant

Table S5: Non-immediate solicited systemic adverse events in children 3-5 years old receiving CoronaVac® or placebo.

Adverse event	CoronaVac® Dose 1 n=181		Placebo Dose 1 n=175		
	**n**	**prop**	**n**	**prop**	**p**
Fever	19	10.5	18	10.3	NS
Fatigue	16	8.84	16	9.14	NS
Anorexia	10	5.52	9	5.14	NS
Diarrhea	10	5.52	7	4	NS
Myalgias	9	4.97	10	5.71	NS
Headache	9	4.97	11	6.29	NS
Vomiting	7	3.87	10	5.71	NS
Mucocutaneous abnormality	4	2.21	7	4	NS
Nausea	2	1.1	0	0	NS
Allergic reaction	1	0.55	1	0.57	NS
	**Dose 2 n=116**		**Dose 2 n=117**		
	**n**	**prop**	**n**	**prop**	**p**
Diarrhea	6	5.17	3	2.56	NS
Vomiting	5	4.31	3	2.56	NS
Anorexia	4	3.45	2	1.71	NS
Fever	3	2.59	5	4.27	NS
Fatigue	2	1.72	3	2.56	NS
Headache	2	1.72	2	1.71	NS
Mucocutaneous abnormality	2	1.72	0	0	NS
Myalgia	1	0.86	2	1.71	NS
Nausea	1	0.86	1	0.85	NS

Prop: proportion; NS: no significant

Table S6: Unsolicited adverse events reported during the study after CoronaVac® dose 1 and 2.

**Adverse event**	**dose 1**	**dose 2**
Respiratory symptoms	237	531
Gastrointestinal symptoms	69	119
Trauma and accidents	10	8
Allergy[a]	23	34
Fever without source of origin	37	48
Miscellaneous	107	129
**Total**	**483**	**869**

[a]allergic rhinitis- decompensate asthma and dermatological symptoms

Table S7: Severe adverse events reported during the study after CoronaVac® dose 1 and 2.

Subject	Gender	Age (years old)	Timing	Event	Outcome	Relationship with study vaccine
Subject 1	F	3	16 days after 1st dose	Febrile illness due to influ-enza and rhinovirus	Recovered	No related
Subject 2	F	5	12 days after 2nd dose	Acute febrile gastroenteritis	Recovered	No related
Subject 3	M	10	12 days after 1st dose	Testicular torsion	Recovered	No related
Subject 4	F	5	4 months and 3 days after 2nd dose	Mesenteric lymphadenitis	Recovered	No related
Subject 5	M	12	4 months and 7days after 2nd dose	Acute gastroenteritis with dehydration	Recovered	No related
Subject 6	M	3	2 months and 23 days after 2nd dose	Bacterial pneumonia	Recovered	No related
Subject 7	M	11	5 months and 23 days after 2nd dose	COVID-19 pneumonia with bacterial superinfection	Recovered	No related
Subject 8	M	5	4 months and 18 days after 2nd dose	Steptococcal Toxic Shock Syndrome	Recovered	No related

Table S8. Other vaccine received during the study.

Vaccine	Age group (years)			
	**3-5**	**6-11**	**12-17**	**Total**
Influenza	734	176	18	928
MMR	149	26	0	175
MenB	37	1	0	38
HPV	0	28	5	33
Varicella	26	1	0	27
Hepatitis A	18	2	1	21
Tdap	0	11	11	22
Hexavalent	5	0	0	5
PPSV23	4	2	0	6
Rabies	1	0	4	5
MenACWY	4	0	0	4
PCV13	2	2	0	4
Yellow Fever	1	0	0	1
Total Vaccines	981	249	39	1269
**Total Subjects**	**558**	**149**	**28**	**735**

SUPPLEMENTARY METHODS:

Inclusion and Exclusion Criteria

Inclusion Criteria

1. Healthy children and adolescents aged 6 months to 17 years;
2. The participants and/or their guardians are able to understand and sign the informed consent voluntarily (in accordance with the local regulations);
3. Able to comply with study procedures based on the assessment of the Investigator;
4. Female participants of childbearing potential (post-menarche girls or in accordance with the local standard of care) may be enrolled in the study if the participant fulfills all the following criteria:

- Has a negative pregnancy test on the day of the first dose (Day 0).
- Has practiced adequate contraception or has abstained from all activities that could result in pregnancy for at least 28 days prior to the first dose (Day 0).
- Has agreed to continue adequate contraception through 3 months following the second dose (Day 28).
- Is not currently breastfeeding.

5. Must be willing to provide verifiable identification (in accordance with the local regulations), has means to be contacted and to contact the investigator during the study.

Exclusion Criteria

Participants are excluded from the study if any of the following criteria apply:

- History of confirmed infection of SARS CoV-2 prior to randomization;
- History of contact with person infected with SARS-CoV-2 (has a positive nucleic acid test or an antigen test) within 14 days prior to randomization;
- Prior administration of an investigational or licensed coronavirus vaccine or current/planned simultaneous participation in another interventional study to prevent or treat COVID-19;

- Allergy to vaccines or vaccine/placebo ingredients, and serious adverse reactions to vaccines, such as urticaria, dyspnea, angioneuroedema;
- Personal or first-grade relative (siblings) history of multisystem inflammatory disease in children (MIS-C);
- Significant chronic illnesses that, in the opinion of the investigator, is at a stage where it might interfere with trial conduct or completion (may include, but are not limited to cardiovascular disease, liver or kidney disorders, respiratory illnesses)
- Significant chronic central nervous system diseases or neuromuscular disorders, psychosis or severe cognitive behavioral disorder, in the opinion of the investigator, including epilepsy, autism spectrum disorder, intellectual disabilities (excluding Down Syndrome);
- Acute central nervous system diseases such as encephalitis/myelitis, acute disseminating encephalomyelitis, and related disorders;
- History of autoimmune and/or haematological diseases (including but not limited to systemic lupus erythematosus, thyroidectomy, autoimmune thyroid disease, any form of malignant tumor, asplenia, functional asplenia, or splenectomy resulting from any condition); well controlled type I diabetes mellitus is allowed;
- History of bleeding disorders (e.g. factor deficiency, coagulopathy or platelet disorder), or prior history of significant bleeding or bruising following IM injections or venipuncture;
- Immunosuppressive therapy (systemic corticoid therapy, e.g. prednisone ≥2 mg/Kg/d or ≥20 mg/day for >14 days), cytotoxic therapy (antineoplastic chemotherapy, radiation therapy), (excluding topical or aerosol corticosteroid therapy) in the past 6 months;
- Receipt of blood products or immunoglobulins in the past 3 months;
- Receipt of other investigational drugs in the past 30 days;
- Receipt of attenuated live vaccines in the past 14 days;
- Receipt of inactivated or subunit vaccines in the past 7 days;

- Emerging of chronic diseases or acute exacerbation of stable chronic diseases (including but not limited to asthma, migraine, gastrointestinal disorder, etc.) prior to randomization;
- Acute febrile illness with oral temperature >37.6°C or axillary temperature >37.4°C on the day of vaccination (refer to section 7.1 Delay/Discontinuation of Study Vaccination); enrollment could be considered if the fever is absent for 72 hours;
- Any confirmed or suspected human immunodeficiency virus (HIV) infection;
- Children in care or under a court order;

According to the investigator's judgment, the subject has any other factors that might interfere with the results of the clinical trial or pose additional risk to the subject due to participation in the study.

Adverse Events of Special Interest (AESI)

The Priority List of Adverse Events of Special Interest in CoronaVac® is included in events of special interest. In this study, the following should be reported expeditiously:

- SAEs;
- Pregnancy in the first 4 weeks after the last vaccination;
- Bell palsy;
- Generalized seizure;
- Guillain-Barré syndrome;
- Acute disseminated encephalomyelitis;
- Hematological thrombocytopenia;
- Immune anaphylaxis;
- Vasculitis;
- MIS-C;
- Other serious local or systemic adverse events after immunization.

COVID-19 case definition:

Definition of RT-PCR confirmed symptomatic COVID-19 (adapted from NMPA standard):

Individuals with at least two type A symptoms, or at least one type B symptom, or chest imaging characteristics, and with a positive RT-PCR test of COVID-19.

1) Clinical symptom

Symptom A (for at least 2 days): Fever, chills, sore throat, tiredness, nasal congestion or running nose, muscle pain, stomachache, headache, nausea or vomiting, diarrhea.

Symptom B: Cough (for at least 2 days), new loss of taste or smell (for at least 2 days), shortness of breath or difficulty breathing (any duration).

2) Chest imaging characteristics of COVID-19: In the early stage, there were multiple small patches and interstitial changes, especially in the lung periphery. Furthermore, it develops into multiple ground glass shadows and infiltrating shadows in both lungs. In severe cases, lung consolidation may occur, and rarely pleural effusion. In MIS-C, heart shadow enlargement and pulmonary edema can be observed in patients with cardiac insufficiency.

Regarding the RT-PCR test, respiratory tract sample (eg, nasopharyngeal swab, throat swab or saliva) will be collected.

Regarding the sensitivity analysis, country specific local case definition also will be adopted.

Members of the PedCoronaVac03CL Study Group:

Center CL01 (Marcoleta, Pontificia Universidad Católica de Chile): Patricio Astudillo Paredes, Héctor Morán Fernández, Javiera Arenas Urra, Stephani Ascui Baeza, María Olivia Cabrera, José Romero Muñoz, Gonzalo Alarcón Andrade, Rocío Rodríguez Espósito, Anwar Gutiérrez Silva and Fernanda Pérez Gutiérrez

Center CL02 (San Carlos de Apoquindo, Pontificia Universidad Católica de Chile): Marcela Potin Santander, Sofia López, Valeria Guerra, Patricia Varela, Daniela Salas and Daniela Ibañez

Center CL03 (Centro médico San Joaquín, Pontificia Universidad Católica de Chile): Macarena Goldsack, Deidyland Arenas Encalada, Andrea Araya Del Pino, Javiera Moore Guerrero, Lorena Pilicita Santana, Vania Valenzuela Guerrero, Catalina Campos Alomar, Mauricio Soto Durán, Bárbara Cortés Miranda, Moira Bravo García, Carolina Mena Valdenegro, Francisca Cortés Espinoza, Adolfo Leiva Urzúa and Lilian Córdova Reyes

Center CL05 (Clínica Alemana, Universidad del Desarrollo): Andrea Schilling, Alberto Trautmann, Ana Fritis, Daniela Pavez, Javiera Arancibia, Lilian Rubio, Paula Viviani, Vinka Basic, and Cassandra Cárcamo

Center CL06 (Clínica Valdivia, Universidad Austral): Mario Calvo Gil, Marisol Wenzel, Nicole Carey, Roberto Burgos, Leonor Gutiérrez and Roxana Martínez

Center CL07 (Hospital de Puerto Montt): Loreto Twele, Daniel Beltrán, Silvana Grandón, Carlos Tovar, Claudia Mella, Gustavo Tripailaf and Pía Jara

Center CL08 (Hospital Gustavo Fricke, Universidad de Valparaíso): Marcela González, Daniela Fuentes, Teresa Ramírez, Mariela Cepeda Corrales, and Nataly Martínez López

Center CL09 (Complejo Asistencial Dr. Sotero del Rio): Valentina Gutiérrez, Felipe Reyes, Armando Lavayen, Melissa González, Monserrat Gutiérrez, Noris Rengifo, Carla Ortega, and Florencia Saber

Center CL10 (Hospital Roberto de Río, Universidad de Chile), Lorena Tapia, Mirta Acuña, Javiera Albornoz, Tania Cariqueo, Alejandro Velásquez, and Yennybeth Leiva Chamorro

Center CL11 (Hospital de niños Dr. Exequiel Gonzalez Cortés, U de Chile): Rodolfo Villena, Paola Flores, Francisca Bartsch, Belén Sepúlveda, Daniela Garmendia, Lorena González, Karen Bastías and Daniela Yévenes

Center CL12 (Hospital Clínico Universidad de Antofagasta, Universidad de Antofagasta): Antonio Cárdenas, Angello Retamal, Carmen Ludeña, Carolina Hermosilla, Gustavo Keilhold, Francisco Cammarata-Scalisi, Jessica Álvarez, Jessica Romero, Pía Villarroel, Francisca Muñoz, Jorge Maya, Andrés Canales, Margarita K. Lay, Christian Muñoz, and Roylester Araya.

Central Laboratory (Laboratorio de Inmunología Traslacional, Laboratorio de Patogénesis Microbiana and Laboratorio de Virus e Inflamación – Facultad de Ciencias Biológicas, Pontificia Universidad Católica de Chile): Alexis M. Kalergis, Pablo A. González, Susan M Bueno, Jorge A. Soto, Felipe Melo-González, Barbara M. Schulz, Roslye Berríos-Rojas, Daniela Rivera-Pérez, Alejandro Piña-Iturbe, Guillermo Hoppe-Elsholz, Luisa F. Duarte, Yaneisi Vázquez, Daniela Moreno-Tapia, Mariana Ríos, Gaspar A. Pacheco, Constanza Méndez, Catalina A. Andrade, Pedro H. Silva, Benjamín Diethelm-Varela, Valentina Pávez, Diane Leighton, Claudia Orellana
